# Case–Control Study of Blood Lead Levels and Attention Deficit Hyperactivity Disorder in Chinese Children

**DOI:** 10.1289/ehp.11400

**Published:** 2008-06-05

**Authors:** Hui-Li Wang, Xiang-Tao Chen, Bin Yang, Fang-Li Ma, Shu Wang, Ming-Liang Tang, Ming-Gao Hao, Di-Yun Ruan

**Affiliations:** 1 School of Life Sciences, University of Science and Technology of China, Hefei, Anhui, People’s Republic of China; 2 Anhui College of Traditional Chinese Medicine, Hefei, Anhui, People’s Republic of China; 3 Anhui Provincial Children’s Hospital, Hefei, Anhui, People’s Republic of China; 4 Nanfang Lee Kum Kee Co., Ltd., Guangzhou, Guangdong, People’s Republic of China; 5 Institute of Anhui Traditional Chinese Medicine, Hefei, Anhui, People’s Republic of China

**Keywords:** attention deficit hyperactivity disorder, blood lead levels, case-control study

## Abstract

**Background:**

Attention deficit/hyperactivity disorder (ADHD) and lead exposure are high-prevalence conditions among children.

**Objective:**

Our goal was to investigate the association between ADHD and blood lead levels (BLLs) in Chinese children, adjusting for known ADHD risk factors and potential confounding variables.

**Methods:**

We conducted a pair-matching case–control study with 630 ADHD cases and 630 non-ADHD controls 4–12 years of age, matched on the same age, sex, and socioeconomic status. The case and control children were systematically evaluated via structured diagnostic interviews, including caregiver interviews, based on the *Diagnostic and Statistical Manual of Mental Disorders*, 4th ed., revised criteria (DSM-IV-R). We evaluated the association between BLLs and ADHD using the Pearson chi-square test for categorical variables and the Student *t*-test for continuous data. We then performed conditional multiple variables logistic regression analyses with backward stepwise selection to predict risk factors for ADHD.

**Results:**

There was a significant difference in BLLs between ADHD cases and controls. ADHD cases were more likely to have been exposed to lead during childhood than the non-ADHD control subjects, with adjustment for other known risk factors [children with BLLs ≥ 10 μg/dL vs. ≤ 5 μg/dL; OR = 6.0; 95% confidence interval (CI) = 4.10–8.77, *p* < 0.01; 5–10 μg/dL vs.≤ 5 μg/dL, OR = 4.9; 95% CI = 3.47–6.98, *p* < 0.01]. These results were not modified by age and sex variables.

**Conclusions:**

This was the largest sample size case–control study to date to study the association between BLLs and ADHD in Chinese children. ADHD may be an additional deleterious outcome of lead exposure during childhood, even when BLLs are < 10 μg/dL.

Attention deficit hyperactivity disorder (ADHD) is one of the most common childhood psychiatric disorders, characterized by developmentally inappropriate levels of inattention, impulsivity, and hyperactivity [American Psychiatric Association (APA) 2000; Biederman and Faraone 2005; Rappley 2005; Remschmidt 2005]. Prevalence of ADHD in children has been reported to be 3–8% worldwide (Froehlich et al. 2007; Leung et al. 1996; Remschmidt 2005). Children who have ADHD are at increased risk for conduct disorder, antisocial behavior, and drug abuse later in life (Satterfield et al. 2007). Moreover, the costs associated with their medical care and education are substantial (Leibson et al. 2001).

Although the causes of ADHD remain unclear, both genetic and environmental factors are thought to influence the etiology of ADHD (Banerjee et al. 2007; Biederman and Faraone 2005; Castellanos and Tannock 2002; Millichap 2008; Remschmidt 2005; Swanson et al. 2007). Family, twin, and adoption studies have demonstrated high heritability, and various polymorphisms of dopamine-related genes have been found to increase susceptibility to ADHD (Castellanos and Tannock 2002; Durston 2003; Khan and Faraone 2006). Furthermore, many environmental risk factors and potential gene–environment interactions have also been shown to increase the risk for the disorder (Banerjee et al. 2007; Biederman and Faraone 2005; Millichap 2008; Thapar et al. 2007). Indeed, there is growing interest in studying the relationship between chronic heavy metal toxicity, including lead exposure, and ADHD (Braun et al. 2006; Konofal and Cortese 2007; Millichap 2008; Nigg et al. 2008).

Lead is one of the well-established environmental poisons, and its general toxic effects, particularly in children, continue to be a major public health issue worldwide [American Academy of Pediatrics (AAP) 2005; Lidsky and Schneider 2003; Needleman 2004]. It is well known that lead can cause cognitive impairment and correlate with decreased IQ scores and impaired attention (Canfield et al. 2003; Koller et al. 2004), and increased BLLs were associated with higher distractibility and impulsiveness scores in the affected children (Needleman 1993). The World Health Organization (WHO) and the U.S. Centers for Disease Control and Prevention (CDC) recommended that child blood lead levels (BLLs) not exceed 10 μg/dL (CDC 1991; WHO 1995). As a result of rapid industrialization in China, it is estimated that tens of millions of children 1–18 years of age have BLLs ≥ 10 μg/dL (Huo et al. 2007; Ren et al. 2006; Wang and Zhang 2006). Furthermore, several recent studies have shown that cognitive deficits and behavioral problems in children still exist even with BLLs < 10 μg/dL (Binns et al. 2007; Braun et al. 2006; Canfield et al. 2003; Koller et al. 2004).

Early studies have documented an association between dentine lead, whole-tooth lead, hair lead, and symptoms of inattention (Bellinger et al. 1994a, 1994b; Fergusson et al. 1993; Needleman and Leviton 1979; Needleman et al. 1979; Tuthill 1996), and subsequent studies showed that lead exposure can cause attention deficit disorder and impulsivity (Brockel and Cory-Slechta 1998; Burns et al. 1999; Eppright et al. 1997; Kahn et al. 1995; Minder et al. 1994; Silva et al. 1988; Thomson et al. 1989; Wasserman et al. 1998, 2001). However, these studies may not be conclusive. First, few studies have investigated the effect of lead exposure on ADHD formally diagnosed with the established criteria in *International Classification of Diseases, 10th Revision* (ICD-10) (WHO 2007) or in Diagnostic and Statistical Manual of Mental Disorders, 4th ed., revised (DSM-IV-R; APA 2000) (Burns et al. 1999; Eppright et al. 1997; Silva et al. 1988). Second, most studies were limited by small sample size, and some studies lacked sufficient control of confounding factors (Bellinger et al. 1994b; Minder et al. 1994). Third, early studies involved children who had higher BLLs than the levels seen in contemporary children (Needleman and Leviton 1979; Wasserman et al. 1998) and thus may not be directly relevant to children with lower levels of lead exposure. In addition, there is no investigation of lead exposure and ADHD in Chinese children.

Therefore, in an effort to evaluate the association between BLLs and the risk for ADHD in Chinese children, we performed a case–control study involving large samples of children 4–12 years of age. We also considered known potential variables in the etiology of ADHD, such as delivery characteristics, perinatal factors, parental psychosocial factors, and prenatal exposure to tobacco and alcohol.

## Methods

### Subjects

This study was designed as a pair-matching case–control study. ADHD subjects were consecutively recruited from children coming for initial or follow-up assessment from October 2003 to August 2007 in two pediatric clinics at the Anhui Provincial Children’s Hospital and the Institute of Anhui Traditional Chinese Medicine in Anhui Province, People’s Republic of China. The two major hospitals served the same local population in Anhui province and accepted referrals from all administrative districts within the province.

All ADHD (ICD-10 codes F90, 208–210) subjects were children of Chinese Han nationality 4–12 years of age at the time of investigation and had a lifetime medical history that fully met the DSM-IV-R criteria (APA 2000; Su et al. 2001) for ADHD. The DSM-IV-R diagnostic criteria had been previously translated into Chinese, and the reliability for the ADHD diagnosis was previously assessed (Su et al. 2001).

The diagnoses of ADHD were derived from a structured diagnostic interview based on *Schedule for Affective Disorders and Schizophrenia for School-Age Children* (K-SADS-E) (Ambrosini 2000), which was modified to assess DSM-IV-R criteria and incorporate parents’ and teachers’ reports of behavioral symptoms, clinical observation of behavior, the Aberrant Behavior Checklist (Aman and Singh 1986), and tests of attention such as the Conners’ Continuous Performance Test (Homack and Riccio 2006). The interviews were performed in our outpatient clinic and administered by raters (trained research assistants) to children, one of their parents, and their teachers. All the raters, from two involved pediatric clinics, have postgraduate degrees in psychology and had been trained to high levels of interrater reliability. We computed κ coefficients of agreement between all the raters and three experienced board-certified child psychiatrists who listened to audio taped interviews made by the raters. Based on 120 interviews, the median κ was 0.87; κ was 0.95 for ADHD. Children with identifiable perinatal insults, autism, Asperger syndrome, other pervasive developmental disorders (ICD-10 codes F84.0–F84.9, 308.0), and mental retardation (ICD-10 codes F70–F79, 312–315) were excluded.

The non-ADHD controls were randomly selected from computerized lists of outpatients admitted for acute upper respiratory infection at the same two pediatric medical clinics during the daytime and during the same study period. By the pair-matched design, each ADHD case and control set had the same sex, the same age (difference between birthdays within 6 months), and almost the same level of socioeconomic status (SES). The controls were given the same full diagnostic assessment as the ADHD cases and screened only for the absence of ADHD without exclusion of any other diagnosis except for the same exclusion criteria applied to cases. SES is measured by poverty-to-income ratio (PIR), and PIR is the ratio of family income to the poverty threshold for the year of the interview. Low SES was defined as having PIR values < 1 in our analysis and high SES as having PIR > 3. The others were regarded as middle SES. The study was approved by our institutional review boards and complied with all applicable requirements of the United States. The parents of all the children in this study provided written informed consent at enrollment.

### Blood sampling and analysis

Blood samples (2 mL/child) were collected in heparinized syringes. Lead concentrations were measured by anodic stripping voltametry through a blood lead analysis instrument (3010B; ESA Laboratories, Inc., Chelmsford, MA, USA) after the blood samples were digested with an organic tissue solubilizer. The limit of detection was 1.0 μg/dL. No detectable values were given values of 0.70 (1.0 divided by the square root of 2). Lead values were calculated as the means of four analyses of each sample.

### Covariates

Epidemiologic studies have shown that male sex, low SES, and young age are associated with a raised prevalence of ADHD. Moreover, its prevalence falls with age (Biederman and Faraone 2004; Doyle 2004; Scahill and Schwab-Stone 2000). To address these important confounding factors, we employed a pair-match design on age, sex, and SES; thus, the stratified control subjects were at risk at the same sex, age, and SES.

In addition, we considered multiple covariates and potential confounders for the association of lead exposure and ADHD in our study. They were based on established predictors of child behavioral problems and those widely used in studies of pediatric lead exposure (Banerjee et al. 2007; Biederman and Faraone 2005; Linnet et al. 2003; Mick et al. 2002; Millichap 2008; St Sauver et al. 2004; Swanson et al. 2007; Wasserman et al. 2001). The following variables were used: family history of ADHD (ADHD in parents and siblings, diagnosed by psychiatrists, obtained from clinical reports), household composition (normal: child lives with biological parents; single: child lives with only one parent; or recombined: child lives in remarried family), maternal tobacco use during pregnancy (at least one cigarette per day during the last trimester), maternal drinking during pregnancy (at least two glasses per week during the entire pregnancy), labor complications, cesarean delivery, perinatal distress [low birth weight and admission to a neonatal intensive care unit (NICU) as markers], parents’ age at childbirth, and parents’ education. Because all the cases recruited were of Chinese Han nationality, the variable of ethnic origin could not be used in our analysis. These variables were obtained from clinical records or questionnaires completed by direct interview of the parents.

Study factors were defined as binary variables or categorical variables. For example, maternal smoking and drinking habits during pregnancy were recorded as binary variables—that is, drinker or nondrinker, cigarette smoker or nonsmoker. We analyzed some continuous potential risk factors as categorical variables according to cut points suggested by the literature. For example, low birth weight is typically defined as < 2,500 g (St Sauver et al. 2004). Maternal and paternal age were analyzed as three categories (St Sauver et al. 2004): < 20 years, 20–30 years, and > 30 years of age. Maternal and paternal education were analyzed as ≤ 9 years of compulsory education, high school education (9–12 years), or some college or advanced training (≥ 12 years).

### Data analysis

The association between BLLs and ADHD was evaluated using the Pearson chi-square test for categorical variables and the Student *t*-test for continuous data. We also performed a conditional logistic regression analysis with a binary outcome of ADHD in relation to BLLs, adjusting for other potential confounding factors (Hosmer and Lemeshow 2000). In the regression model, BLLs were analyzed as an ordered categorical variable and recorded as three categories: ≤ 5 μg/dL, 5–10 μg/dL, and ≥ 10 μg/dL. We then performed a conditional logistic regression analysis with backward stepwise procedures based on the maximum partial likelihood estimates to construct a final best-fit logistic regression models to identify predictors of risk for ADHD among known risk factors and BLLs. We estimated odds ratios (ORs) and 95% confidence intervals (CIs) for differing levels of exposure. All statistical tests were considered to be significant at an alpha level of 0.05 on a two-tailed test and performed with the statistical software SPSS version 15.0 (SPSS Inc., Chicago, IL, USA).

## Results

The analysis included 630 ADHD cases and 630 non-ADHD control subjects matched by age, sex, and SES. There were 434 sets of boys and 196 sets of girls. The average ages were 7.9 ± 2.1 years. The mean BLLs were 8.77 ± 3.89 μg/dL in the ADHD group and 5.76 ± 3.39 μg/dL in the control group. The ADHD group had higher BLLs (*p* < 0.05, see Table 1). There was no significant difference in BLLs between males and females.

**Figure 1:**
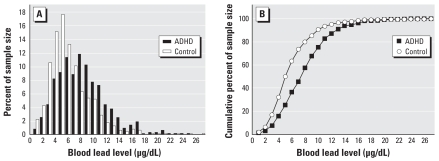
(*A*) Blood lead distribution (%) of ADHD cases and controls. (*B*) Cumulative distribution (%) of blood lead for ADHD cases and controls.

Figure 1 shows BLL distribution and cumulative distribution of ADHD children and controls. Only 10.1% of non-ADHD children had BLLs > 10 μg/dL, whereas this percentage increased significantly to 24.4% in ADHD children. (χ^2^ = 237, *p* < 0.01). In addition, 49.8% of non-ADHD children had BLLs > 5 μg/dL, whereas of the ADHD cases, 74.7% had BLLS > 5 μg/dL (χ^2^ = 116, *p* < 0.01).

Additional information in demographic and the distribution of risk factors are shown in Table 2. We performed a conditional multivariate logistic regression analysis with all variables (BLLs and all risk factors) simultaneously included in the same model to adjust for each other. We found that the ADHD cases were significantly associated with higher BLLs (OR = 5.19, *p* < 0.01 for children with BLLs 5–10 μg/dL; OR = 7.15, *p* < 0.01 for with BLLs ≥ 10 μg/dL, using the sample with BLLs ≤ 5 μg/dL as referent) and family history of ADHD (OR = 4.54, *p* < 0.01, compared with the sample without familial ADHD history). The risk for ADHD decreased as the mother’s education level increased (OR = 0.69, *p* = 0.017, using the sample of ≤ 9 years maternal education as referent). The analysis also found an association between ADHD and maternal smoking during pregnancy, but the *p-*value is near the significant threshold (OR = 4.04, *p* = 0.047). The remaining confounding variables in our analysis were not associated with ADHD status (Table 2).

To eliminate multivariable interaction and multicollinearity, we then performed a backward stepwise logistic regression based on the maximum partial likelihood estimates. In the final best-fit model, the association between ADHD and maternal smoking during pregnancy was excluded, but ORs for the associations between ADHD and family history of ADHD, maternal education levels, and BLLs were largely unchanged from estimates obtained in the original model used in Table 2 (Table 3).

We also performed a conditional multivariate stepwise logistic regression analysis stratified by sex and age. As with the total sample, ADHD cases were significantly associated with higher BLLs than the lower BLLs in all subdefinitions (Table 4), which indicates that increased risk for ADHD associated with BLLs is not modified by age and sex.

## Discussion

We examined the effect of BLLs in ADHD cases and non-ADHD controls with adjustment for most other known risk factors in Chinese children 4–12 years of age. We found ADHD cases were more likely to have been exposed to lead. The children with BLLs > 10 μg/dL had 4.1- to 8.7-fold higher risk for ADHD. Even when their BLLs were under the recommended level but > 5 μg/dL, the risk also showed a 3.5- to 7.0-fold increase. Family history of ADHD was another positive high-risk factor. Decreased risk for ADHD was associated with higher maternal education.

Lead may play important roles in the etiology of ADHD. Recently, Braun et al. (2006) analyzed data from a U.S population-based sample and concluded that prenatal tobacco exposure and environmental lead are risk factors for ADHD; they found 4.1-fold increased odds of ADHD with increased BLLs. Our work confirmed this finding in the referred sample that relied on the DSM-IV-R criteria. In addition, Nigg et al. (2008) reported that low-level lead exposure was associated with formal clinical diagnostic ADHD by DSM-IV-R criteria in a community sample in the United States, where regulations have reduced the incidence of lead poisoning and the current population average of lead levels is relatively lower. This is consistent with our finding that the risk of ADHD was associated with low lead exposures (< 10 μg/dL) in Chinese children. Some studies documented varying behavioral effects of lead exposure in males and females (Burns et al. 1999; Ris et al. 2004). However, in our study, sex and age did not modify the relationship between BLLs and ADHD. Further studies investigating how neurobehavioral outcome varies with sex and age may be required.

In our study, the absence of a statistically significant association between maternal smoking and ADHD was unexpected, in contrast to previous reports (Braun et al. 2006; Linnet et al. 2003; Swanson et al. 2007). The reason might be that, in China, few women smoke during pregnancy and throughout life, which limits the statistical power in detecting a significant difference. Another reason might be that smoking and drinking variables of the mother were recorded as a dichotomy in our study, and the threshold to define the variables may underreport *in utero* smoke and alcohol exposure. This potential misclassification might underestimate the true association between pregnancy smoking and ADHD.

Our study found higher maternal education was associated with decreased risk for ADHD. This result is consistent with previous work, which indicated that low maternal education, low SES, and single parenthood are important adverse factors for ADHD (Biederman and Faraone 2005; Millichap 2008; St Sauver et al. 2004). Apart from environmental risk factors for ADHD, our current report indicated that children with a family history of ADHD were more likely to be diagnosed with ADHD. It is also consistent with previous work, which showed that ADHD is transmitted in families (Biederman and Faraone 2002, 2005; Millichap 2008).

Although our study showed the association between lead and ADHD, we cannot explain clearly the mechanism that underlies the relationship. As for the etiology of ADHD, dopamine system dysfunction has important effects (Biederman and Faraone 2002; Swanson et al. 2007). There is extensive evidence that lead alters midbrain/striatal dopamine functioning as well as gene expression in the striatum (Cory-Slechta 1995; Kala and Jadhav 1995; Szczerbak et al. 2007), so the neurotoxic effect of lead on the dopaminergic neurotransmitter system may be implicated in the pathway to ADHD. Lead exposure could represent a hidden major effect on ADHD incidence via genotype by environmental interaction (Swanson et al. 2007).

### Strengths of the study

The strengths of this study are as follows: *a*) Our sample size was the largest to date in case–control studies to investigate BLLs and ADHD. *b*) The ADHD diagnosis was made through an extensive clinical evaluation based on the DSM-IV-R diagnostic instrument and performed by child and adolescent psychiatrists. *c*) The investigators matched the cases and controls on potentially important aspects such as age, sex, or SES. This is important because lead levels are highest among younger children compared with adolescents, and ADHD is of higher prevalence among children of elementary and middle-school age.

### Clinical implications

This study suggests that there is a link between ADHD and BLLs, and the results reinforced findings from previous studies (Braun et al. 2006; Nigg et al. 2008). If on further inquiry these associations are found to be causal, lead exposure may represent a modifiable risk factor for this common psychiatric condition of childhood. Considering that ADHD typically starts in early childhood and that lead poisoning is one of the most common and entirely preventable pediatric problems (AAP 2005; CDC 1991), strategies in public health must focus on practicing primary and secondary prevention of lead exposure in children. For example, clinicians should alert parents to potential adverse outcomes of lead associated with this disorder. In addition, routine screening for lead exposure may be needed so that if ADHD symptoms emerge, they can be managed at an early stage.

Moreover, BLLs < 10 μg/dL also indicated a risk factor. This may suggest that the lower standard may effectively protect children from the harmful effects of lead, which is consistent with previous investigations (AAP 2005; Canfield et al. 2003; Surkan et al. 2007).

Some studies (Ruff et al. 1993; Tong et al. 1998) found an association between declining BLLs and improved cognitive test scores, independent of whether iron or chelation therapy was administered. However, other works (AAP 2005; Canfield et al. 2003) indicated that the damage caused by blood lead exposure is irreversible. Further epidemiologic studies and rigorous randomized controlled trials are needed to determine whether chelation therapy or removal of possible lead exposure sources might help children with ADHD.

### Limitations

This study has several limitations. First, temporality between high BLLs and ADHD could not be ascertained definitely in this case–control design. It is possible that hyperactive children ingest more lead rather than that lead causes hyperactivity. Therefore, further studies with serial lead measurements, even from the antenatal to the postnatal period, and more continuous measures of ADHD symptoms or of specific underlying neurobehavioral domains may be required to document the temporal relationship between lead exposure and development of ADHD. Second, some might argue that concurrent blood lead tests are not an adequate biomarker of a child’s lifetime exposure. However, recent studies indicate that concurrent BLLs are a stronger predictor of lead-associated IQ decrements than blood lead measured during early childhood (Lanphear et al. 2005). Third, recall bias is one of the problems of the case–control study. In this study, the main variable of interest was the BLLs, which were objectively measured. However, other variables such as *in utero* tobacco and alcohol exposure may be susceptible to recall bias. Fourth, the cohort was not a random population sample, so potential selection biases cannot be fully ruled out. In addition, because all our sample was Chinese Han, no children living in foster families were included, and none of the sample had health insurance, our results may not generalize to children with different socioeconomic or ethnic backgrounds. Finally, it is interesting that Nigg et al. (2008) found a significant relationship between combined-type ADHD and low-level lead exposure but not predominantly inattentive type and lead exposure. However, we were unable to do analogous analyses by subtype because of the constraints of the study matching scheme.

## Conclusions

This hospital-based case–control study suggests a strong relationship exists between ADHD and lead exposure, even low-level lead exposure (< 10 μg/dL BLL), in Chinese children. If this finding is confirmed in future studies, the potential to prevent ADHD by reducing childhood lead exposure should be considered.

## Figures and Tables

**Table 1:** Mean values of BLL of ADHD cases and controls.

	BLL (μg/dL) (mean ± SE)				
Group	Total (*n* = 630)	Male (*n* = 434)	Female (*n* = 196)	Percent > 5 μg/dL	Percent > 10 μg/dL
ADHD	8.77 ± 3.89	8.89 ± 4.0	8.67 ± 4.17	75.8	24.4
Control	5.76 ± 3.39	5.86 ± 3.44	5.55 ± 3.21	49.8	10.1
*t*-Value, χ^2^	*t* = 14.6	*t* = 11.96	*t* = 8.30	χ^2^ = 237	χ^2^ = 116
*p*-Value	< 0.05	< 0.05	< 0.05	< 0.01	< 0.01

**Table 2:** Demographic and distribution of risk factors of ADHD cases and controls.

Characteristic	ADHD (*n* = 630)	Controls (*n* = 630)	OR^a^	*p-*Value^a^
Matched factors				
Age (years)				
4–6	172	172	[Table-fn tfn1-ehp-116-1401]—	[Table-fn tfn1-ehp-116-1401]—
7–9	303	303		
10–12	155	155		
Sex				
Male	434	434	[Table-fn tfn1-ehp-116-1401]—	[Table-fn tfn1-ehp-116-1401]—
Female	196	196		
SES (PIR)				
≤ 1	58	58	[Table-fn tfn1-ehp-116-1401]—	[Table-fn tfn1-ehp-116-1401]—
1–3	436	436		
≥ 3	136	136		
Child factors				
Blood lead (μg/dL)				
≤ 5	101	316		
5–10	326	255	5.19	< 0.01
≥ 10	203	59	7.15	< 0.01
Household composition				
Two parent	Referent	Referent		
Single parent	27	23	1.05	0.89
Recombined	15	17	0.50	0.11
Low birth weight (< 2,500 g)	55	64	0.68	0.13
Twin	4	10	0.29	0.08
Family history of ADHD	21	4	4.54	0.02
Pregnancy, labor, or delivery characteristics				
Labor or delivery complications	58	70	0.71	0.16
Surgical procedure required	59	67	0.63	0.06
Premature labor	27	22	0.84	0.65
NICU required	39	45	0.57	0.08
Parental factors				
Age of mother (years)				
≤ 20	81	65		
20–30	461	459		
≥30	88	106	0.92	0.75
Age of father (years)				
≤ 20	20	16		
20–30	479	493		
≥ 30	131	121	1.15	0.41
Maternal education (years)				
≤ 9	154	127		
9–12	393	371		
≥ 12	83	132	0.69	0.017
Paternal education (years)				
≤ 9	123	103		
9–12	373	376		
≥12	134	151	1.07	0.97
Maternal drinking during pregnancy	11	6	1.20	0.77
Maternal smoking during pregnancy	6	9	4.04	0.047

—, no data.

a Obtained from the multivariate logistic regression model that simultaneously included all the risk factors and the BLLs.

**Table 3:** Risk factors identified in stepwise logistic regression model.^a^

Variables	β^b^	SE	Wald test	*p*-Value	OR^c^ (95% CI)
BLL					
≤ 5					
5–10	1.59	0.18	79.86	< 0.01	4.92 (3.47–6.98)
≥10	1.79	0.19	85.79	< 0.01	6.00 (4.11–8.77)
Family history of ADHD	1.73	0.63	7.51	< 0.01	5.65 (1.64–19.46)
Maternal education (years)					
≤9					
9–12	−0.13	0.17	0.60	0.438	0.88 (0.63–1.23)
≥ 12	−0.49	0.19	6.90	< 0.01	0.62 (0.43–0.88)

a Variable(s) entered on step 1: BLLs, household composition, birth weight, twin, family history of ADHD, labor complications, cesarean, premature labor, NICU, mother’s age, father’s age, maternal education, paternal education, prenatal tobacco exposure and prenatal alcohol exposure.

b βvalues are the estimated unstandardized regression coefficients.

c OR indicates likelihood of an ADHD.

**Table 4:** Increased risks for ADHD associated with BLLs in different sample definitions.

BLL (μg/dL)	OR (95% CI)	*p-*Value
Total sample (*n* = 1,260)		
≤ 5	1	—
5–10	4.92 (3.47–6.98)	< 0.01
≥ 10	6.00 (4.11–8.77)	< 0.01
Male sample (*n* = 868)		
≤ 5	1	—
5–10	4.49 (2.97–6.80)	< 0.01
≥ 10	6.69 (4.20–10.67)	< 0.01
Female sample (*n* = 392)		
≤ 5	1	—
5–10	5.62 (2.79–11.0)	< 0.01
≥ 10	7.38 (3.66–14.88)	< 0.01
Age 4–6 years sample (*n* = 344)		
≤ 5	1	—
5–10	6.86 (3.17–14.86)	< 0.01
≥ 10	13.41 (5.04–35.69)	< 0.01
Age 7–9 years sample (*n* = 606)		
≤ 5	1	—
5–10	5.78 (3.33–10.00)	< 0.01
≥ 10	8.53 (4.72–15.422)	< 0.01
Age 10–12 years sample (*n* = 310)		
≤ 5	1	—
5–10	3.89 (1.82–8.32)	< 0.01
≥ 10	4.13 (2.01–8.49)	< 0.01

This table is obtained from the multivariate logistic regression model that simultaneously included all the risk factors and the BLL. OR indicates likelihood of ADHD.
